# First description of the male of *Oecobiusprzewalskyi* Hu & Li, 1987 (Araneae, Oecobiidae) from Shigatse City, Tibet, China

**DOI:** 10.3897/BDJ.11.e112801

**Published:** 2023-10-25

**Authors:** Changhao Hu, Jie Liu, Kai Wang

**Affiliations:** 1 The State Key Laboratory of Biocatalysis and Enzyme Engineering of China, College of Life Science, Hubei University, Wuhan, China The State Key Laboratory of Biocatalysis and Enzyme Engineering of China, College of Life Science, Hubei University Wuhan China; 2 Hubei Key Laboratory of Regional Development and Environmental Response, Faculty of Resources and Environmental Science, Hubei University, Wuhan, China Hubei Key Laboratory of Regional Development and Environmental Response, Faculty of Resources and Environmental Science, Hubei University Wuhan China; 3 School of Nuclear Technology and Chemistry & Biology, Hubei University of Science and Technology, Xianning, China School of Nuclear Technology and Chemistry & Biology, Hubei University of Science and Technology Xianning China

**Keywords:** Qinghai-Tibet Plateau, morphology, taxonomy, biodiversity

## Abstract

**Background:**

With 90 described species, the genus *Oecobius* Lucas, 1846 is the largest genus of the family Oecobiidae Blackwall, 1862, five of which are known from China. Since *Oceobiusprzewalskyi* was described by Hu & Li in 1987, no males of this species have ever been reported.

**New information:**

The male of *Oceobiusprzewalskyi* is described for the first time, based on the specimens collected in Tibet Autonomous Region. Morphological description and illustrations are given.

## Introduction

The genus *Oecobius* Lucas, 1846 is distributed in Asia, Europe, Africa, North and South America with 90 species, five of them recorded in China: *O.cellariorum* (Dugès, 1836), *O.marathaus* Tikader, 1962, *O.nadiae* (Spassky, 1936), *O.navus* Blackwall, 1859 and *O.przewalskyi* Hu & Li, 1987 (World Spider Catalog 2023). *Oecobius* species are usually small spiders (< 4 mm), which build sparse webs in the corners or gaps of houses and hide themselves in webs (Zhang and Wang 2017).

The species *Oecobiusprzewalskyi* Hu & Li, 1987 was firstly described, based on female specimens only from Shigatse (Tibet Autonomous Region, China) (Hu and Li 1987). Recently, the authors examined specimens collected from the type locality and found that one male and one female seemed to be this species, based on comparison with the illustration from Hu and Li (1987). Based on the currently-known specimens, this species is only distributed in Shigatse City, Tibet. The aim of the current paper is to report the male for the first time and re-describe the female, with provision of detailed morphological photos.

## Materials and methods

The specimens examined in this study were deposited in the Centre for Behavioural Ecology and Evolution, College of Life Sciences, Hubei University in Wuhan. Specimens were examined using an OLYMPUS SZX7 stereomicroscope. Photographs were taken with a LEICA M205 C stereomicroscope and OLYMPUS SXZ16 microscope and final multifocal images were produced with Helicon Focus (Version 7.7.0). The male palp was examined and photographed after dissection. The epigyne was examined after being dissected from the spider’s body. The epigyne was removed and treated in a warmed 0.1 mg/ml Protease K solution before study. All morphological measurements were calculated using a Leica M205 C stereomicroscope. Eye diameters were taken at the widest point. Leg measurements were given as total length of leg (femur, patella, tibia, metatarsus, tarsus). All measurements were in millimetres (mm).

Morphological terminology follows Coddington (1990) and Magalhães and Santos (2018) for male palps. Abbreviations: **ALE** anterior lateral eyes, **AME** anterior median eyes, **CD** copulatory duct, **CO** copulatory opening, **E** embolus, **EA** embolic apophysis, **I, II, III, IV** legs I to IV, **PLE** posterior lateral eyes, **PME** posterior median eyes, **Sp** spermatheca, **TA** tegular apophysis, **TL I** tegular lobe I.

## Taxon treatments

### Oecobius przewalskyi

Hu & Li, 1987

A7F0A249-EF01-584B-B700-056F80A7E42F

https://www.gbif.org/species/2162478

Oecobius przewalskyi Hu & Li, 1987 in Hu and Li (1987): 247, figs. 1.1‒3; Song et al. (1999): 77, figs. 31J‒K; Hu (2001): 89, figs. 12.1‒3.

#### Materials

- **Type status:** Holotype. **Occurrence:** occurrenceRemarks: not examined; recordedBy: Aihua Li; individualCount: 1; sex: female; lifeStage: adult; **Location:** continent: Asia; country: China; countryCode: China/CN; stateProvince: Tibet; verbatimLocality: Shigatse; verbatimElevation: 3800 m; **Event:** year: 1985; month: 7; day: 31
- **Type status:** Paratype. **Occurrence:** occurrenceRemarks: not examined; recordedBy: Aihua Li; individualCount: 4; sex: 4 females; lifeStage: adult; **Location:** continent: Asia; country: China; countryCode: China/CN; stateProvince: Tibet; verbatimLocality: Shigatse; verbatimElevation: 3800 m; **Event:** year: 1985; month: 7; day: 31
- **Type status:** Other material. **Occurrence:** recordedBy: Fengxiang Liu; individualCount: 2; sex: 1 male, 1 female; lifeStage: adult; **Location:** continent: Asia; country: China; countryCode: China/CN; stateProvince: Tibet; verbatimLocality: Shigatse, Mountain of Tashilhunpo Monastery; verbatimElevation: 3836 m; verbatimLatitude: 29.265392°N; verbatimLongitude: 88.871941°E; **Event:** year: 2002; month: 9; day: 6

#### Description

**Figure 1.**
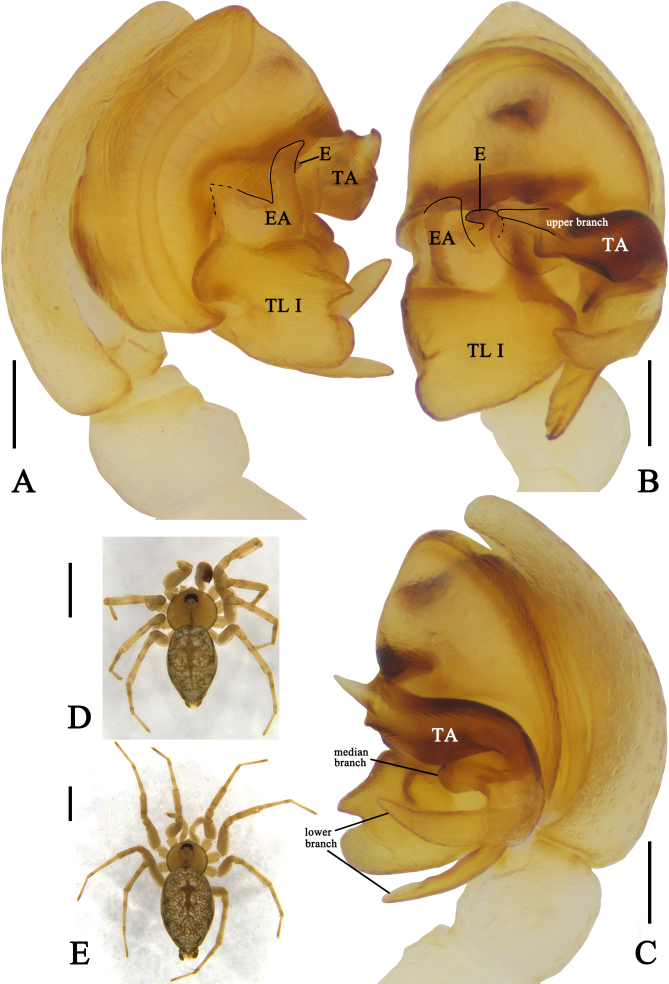
*Oecobiusprzewalskyi* Hu & Li, 1987 from Shigatse: **A** Left male palp, prolateral view; **B** Same, ventral view; **C** Same, retrolateral view; **D** Habitus in dorsal view, male; **E** Same, female. Abbreviations: E‒embolus, EA‒embolic apophysis, TA‒tegular apophysis, TL I‒tegular lobe I. Scale bars: 0.1 mm (A‒C), 1 mm (D‒E).

**Male**: Total length 2.18. Carapace 0.80 long, 0.93 wide. Abdomen 1.49 long, 0.95 wide. Diameters of eyes: AME 0.04, ALE 0.06, PME 0.05, PLE 0.06. Interdistances of eyes: AME‒AME 0.04, AME‒ALE 0.03, PME‒PME 0.08, PME‒PLE 0.01, AME‒PME 0.06, ALE‒PLE 0.02. Eyes white with black rings, except PME. Carapace light yellowish-brown with black margin, except clypeal projection, fovea black. Palps, chelicerae, labium and legs pale brown without marks. Measurements of legs: I 2.81 (0.83, 0.25, 0.67, 0.54, 0.52), II 3.00 (0.85, 0.24, 0.72, 0.69, 0.50), III 3.20 (0.92, 0.25, 0.73, 0.77, 0.53), IV 3.30 (0.92, 0.22, 0.76, 0.86, 0.54). Leg formula: IV-III-II-I. Abdomen dorsally yellowish-brown with white irregular marks, cardiac mark light brown. Abdomen ventrally light yellow with few white irregular marks (Fig. 1D).

Palp as in diagnosis. Embolus (E) short and small, located in the median of bulb in ventral view and bent prolaterally; tegular apophysis (TA) four-branched, upper branch strongly sclerotic, with curled end and an outside tip, median branch small and rounded, two lower branches horn-like; embolic apophysis (EA) point to upward side of bulb and with serrated margin, dorsal embolic apophysis (EA) covered; tegular lobe I (TL I) large and thick, with two apophyses, upper apophysis pointed and lower apophysis rounded (Fig. 1A‒C).

**Female**: Total length 3.40. Carapace 1.03 long, 1.20 wide. Abdomen 2.57 long, 1.59 wide. Diameters of eyes: AME 0.08, ALE 0.07, PME 0.06, PLE 0.06. Interdistances of eyes: AME‒AME 0.10, AME‒ALE 0.04, PME‒PME 0.10, PME‒PLE 0.02, AME‒PME 0.01, ALE‒PLE 0.02. Measurements of legs: I 4.05 (1.24, 0.28, 0.93, 0.88, 0.72), II 4.44 (1.30, 0.37, 1.04, 1.02, 0.71), III 4.41 (1.28, 0.33, 1.01, 1.10, 0.69), IV 4.58 (1.30, 0.38, 1.09, 1.21, 0.60). Leg formula: IV-II-III-I. Colouration as in male, except cardiac mark dark brown, abdomen ventrally light yellow with white irregular marks (Fig. 1E).

**Figure 2.**
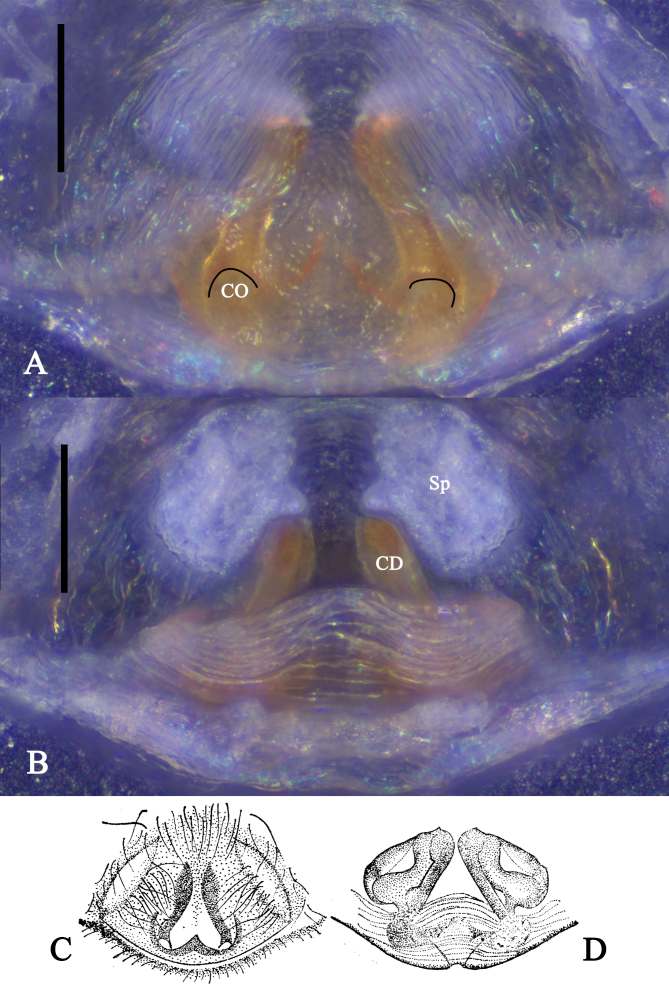
*Oecobiusprzewalskyi* Hu & Li, 1987 female from Shigatse: **A** Epigyne, ventral view; **B** Vulva, dorsal view; **C** Epigyne from the original paper, ventral view (Hu and Li 1987); **D** Vulva from the original paper, dorsal view (Hu and Li 1987). Abbreviations: CD‒copulatory duct, CO‒copulatory opening, Sp‒spermatheca. Scale bars: 0.1 mm (A‒B).

Copulatory organ as in diagnosis. Epigyne without process and scape. Copulatory openings (CO) located in posterior side; copulatory ducts (CD) visible in ventral view; vulva with wrinkles in posterior side, left copulatory duct (CD) pointing to 1 o'clock; spermathecae (Sp) oval and membranous, located in anterior side (Fig. 2).

#### Diagnosis

Male of *Oecobiusprzewalskyi* can be distinguished from other *Oecobius* species by 1. tegular apophysis (TA) four-branched, upper branch with curled end and an outside tip, median branch small and rounded, two lower branches horn-like; 2. embolic apophysis (EA) with serrated margin; 3. tegular lobe I (TL I) thick, with two apophyses, upper apophysis pointed and lower apophysis rounded (Fig. 1A‒C). Female of *O.przewalskyi* can be distinguished from the other species of *Oecobius* by 1. epigyne without process and scape; 2. copulatory ducts (CD) visible in ventral view, left copulatory duct (CD) pointing to 1 o'clock (Fig. 2).

#### Distribution

**Figure 3.**
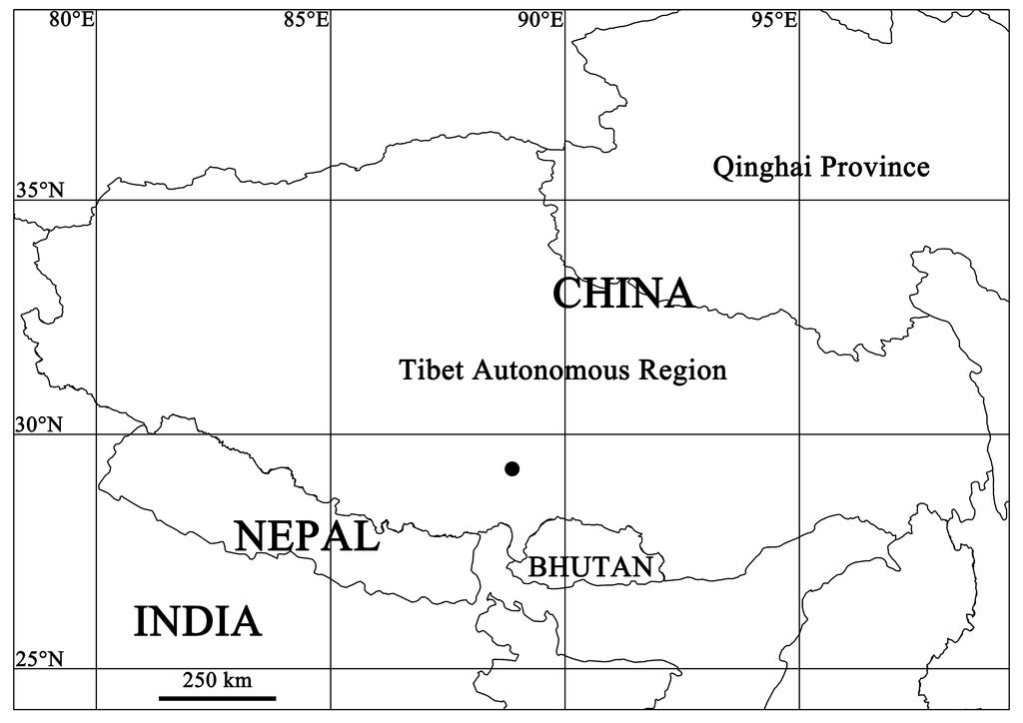
Collection locality and type locality of *Oecobiusprzewalskyi* Hu & Li, 1987 in Tibet, China.

China (Tibet) (Fig. 3).

#### Notes

Although we did not examine the type specimens of *O.przewalskyi*, the left copulatory duct (CD) which is pointing to 1 o'clock and the two oval spermathecae (Sp) shown in the original illustrations (Fig. 2) leave no doubts that our identification is correct.

## Supplementary Material

XML Treatment for Oecobius przewalskyi
